# Distinct Brain Dynamic Functional Connectivity Patterns in Schizophrenia Patients With and Without Auditory Verbal Hallucinations

**DOI:** 10.3389/fnhum.2022.838181

**Published:** 2022-04-07

**Authors:** Yao Zhang, Jia Wang, Xin Lin, Min Yang, Shun Qi, Yuhan Wang, Wei Liang, Huijie Lu, Yan Zhang, Wensheng Zhai, Wanting Hao, Yang Cao, Peng Huang, Jianying Guo, Xuehui Hu, Xia Zhu

**Affiliations:** ^1^Military Medical Center, Xijing Hospital, Fourth Military Medical University, Xi’an, China; ^2^School of Biomedical Engineering, Fourth Military Medical University, Xi’an, China; ^3^Fundamentals Department, Air Force Engineering University, Xi’an, China; ^4^Department of Radiology, Fourth Military Medical University, Xi’an, China; ^5^School of Basic Medicine, Fourth Military Medical University, Xi’an, China; ^6^Department of Medical Psychology, Fourth Military Medical University, Xi’an, China; ^7^Department of Nursing, Xijing Hospital, Fourth Military Medical University, Xi’an, China

**Keywords:** schizophrenia, auditory verbal hallucinations, dynamic functional connectivity, static functional connectivity, k-means clustering

## Abstract

Schizophrenia patients with auditory verbal hallucinations (AVHs) are diseased groups of serious psychosis with still unknown etiology. The aim of this research was to identify the neurophysiological correlates of auditory verbal hallucinations. Revealing the neural correlates of auditory hallucination is not merely of great clinical significance, but it is also quite essential to study the pathophysiological correlates of schizophrenia. In this study, 25 Schizophrenia patients with AVHs (AVHs group, 23.2 ± 5.35 years), 52 Schizophrenia patients without AVHs (non-AVHs group, 25.79 ± 5.63 years) and 28 healthy subjects (NC group, 26.14 ± 5.45 years) were enrolled. Dynamic functional connectivity was studied with a sliding-window method and functional connectivity states were then obtained with the k-means clustering algorithm in the three groups. We found that schizophrenia patients with AVHs were characterized by significant decreased static functional connectivity and enhanced variability of dynamic functional connectivity (non-parametric permutation test, Bonferroni correction, *p* < 0.05). In addition, the AVHs group also demonstrated increased number of brain states, suggesting brain dynamics enhanced in these patients compared with the non-AVHs group. Our findings suggested that there were abnormalities in the connection of brain language regions in auditory verbal hallucinations. It appears that the interruption of connectivity from the language region might be critical to the pathological basis of AVHs.

## Introduction

Schizophrenia is a mental illness, but its harm has been seriously underestimated due to its low incidence and small number of direct deaths. In fact, schizophrenia is highly disabling, has a significant impact on families and society, and is prone to relapse. Clinically, it is often manifested as different syndromes with different symptoms, involving various disorders in perception, thinking, emotion, behavior and other aspects, as well as the disharmony between psychological activities and the environment. Among the above-mentioned symptoms, auditory verbal hallucinations probably occurred among 60–80% of schizophrenia patients (Saha et al., 2005; Petrolini et al., 2020; Sun et al., 2021). Auditory verbal hallucinations (AVHs) is one of the main symptoms of schizophrenia and serves as an important clinical index for the diagnosis of schizophrenia. It refers to hearing without corresponding external sound stimulation acting on the auditory organs. Twenty-five percent of auditory verbal hallucinations patients are chronic and difficult to cure (Allen et al., 2012; Koops et al., 2016). It has been confirmed that schizophrenia patients have structural abnormalities of the brain, but the nature of abnormalities is not consistent (Cui et al., 2017b; Nakahara et al., 2018; Wei Y. et al., 2018).

The content of AVHs often involves threatening or commanding to the patient, or talking about the patient’s thoughts, or commenting on the patient’s behavior, which brings great mental suffering to the patient (Garwood et al., 2015). Especially when under the control of commanding auditory hallucinations, patients may break out strong aggressive or destructive behaviors, thus endangering themselves, families or even the social surroundings due to some sudden self-injury or wounding violence. Auditory verbal hallucinations in schizophrenia are serious psychosis with still unknown etiology. Although after more than 20 years of neuroimaging research, people still do not know the neurophysiological mechanism of auditory verbal hallucinations. Revealing the neural mechanism of AVHs is not only of great clinical significance, but also of great importance as to explore the pathophysiological mechanism of schizophrenia. In recent years, various neuroimaging technologies have been used in the research related to AVHs, and many breakthroughs have been made, which not only provide important bases for the diagnosis and treatment of schizophrenia with AVHs, but also provide vital support in revealing the mechanism of AVHs. Through a series of studies, the neural mechanism of AVHs has also made fruitful progresses. It is found that AVHs are highly correlated with the structural and functional changes in brain regions related to speech generation and perception (Barber et al., 2021). Relevant research results have proved that the formation of AVHs is mainly related to the left middle temporal gyrus, the left temporal parietal lobe, and the left inferior frontal lobe (Zhang et al., 2017; Zhuo et al., 2021). Recent studies have also shown that the cortical thickness in the frontal and temporal cortical areas of patients with AVHs is thinner. Voxel-based morphological studies reported that the severity of auditory hallucinations was associated with the temporal lobe, including the primary and secondary auditory cortex. FMRI studies found that AVHs were related to the over activation of left inferior frontal cortex and left middle temporal cortex. Meta analysis also showed that AVHs were highly correlated with left inferior frontal gyrus and left inferior parietal lobe (Kühn and Gallinat, 2012; Cui et al., 2016, 2018).

The cause of auditory hallucination is much more complex than regional brain abnormalities. Many neuroimaging studies suggest that cognitive dysfunction could not simply be attributed to the structural lesions and functional disorders in a single brain region or several brain regions (Karnath et al., 2018; Herbet and Duffau, 2020). Cognitive dysfunction is often caused by abnormal connections between brain regions. Therefore, it is not enough just to understand the pathogenesis of auditory hallucinations by merely studying the abnormalities in specific brain regions of schizophrenia patients with AVHs (Diederen et al., 2012; Sehatpour et al., 2020).

Most fMRI studies (Chang et al., 2017; Mallikarjun et al., 2018; Geng et al., 2020) investigating functional connectivity (FC) in schizophrenia and hallucinations have employed a static connectivity approach, whereby FC is averaged over scan time. There is substantial evidence for abnormal FC in schizophrenia, but findings vary widely. Dynamic functional connectivity is an extension of traditional static functional connectivity, as this analysis allows exploration of temporal changes in connectivity. Weber et al. (2020) found that hallucination severity did not show a significant relationship with dynamic FC.

Although previous studies have shown that the emergence of AVHs is related to the changes in the connectivity of brain language networks, there are only a few systematic studies on how the changes in the structural and functional connections of brain language regions in schizophrenia patients lead to AVHs, as well as whether these abnormalities have their internal relationship. In this study, static connectivity analysis and dynamic connectivity analysis were complemented. We believe that studying the connectivity of brain language network might shed light on the revelation of the neuropathological mechanism of auditory hallucinations, which is also of great significance in the diagnosis and treatment of schizophrenia patients with auditory verbal hallucinations.

## Materials and Methods

### Participants

Twenty-five schizophrenia patients with auditory verbal hallucinations, 52 schizophrenia patients without auditory verbal hallucinations and 28 healthy subjects were enrolled. All subjects participated in the experiment voluntarily and signed the informed consent form before the experiment. Two weeks before the scan, the patients stopped taking psychotropic drugs. The experiment was approved by the Ethics Committee of the Fourth Military Medical University. All patients were diagnosed by two senior clinical psychiatrists in the Department of Psychosomatics, Xijing Hospital of the Fourth Military Medical University according to DSM-IV standards. Participants’ information is shown in Table 1.

**TABLE 1:** Demographic data of the three groups.

	AVHs	Non-AVHs	NC	p
No.	25	52	28	–
Age	23.20	25.79	26.14	0.06
Gender (male/female)	15/10	28/24	15/13	0.283

*AVHs, Schizophrenia patients with auditory verbal hallucinations; non-AVHs, Schizophrenia patients without auditory verbal hallucinations; NC, Healthy subjects without schizophrenia.*

### Data Acquisition

The MRI data were collected from a 3.0-Tesla SIEMENS Magnetom Trio Tim scanner in Xijing Hospital. Resting-state fMRI images were acquired with an echo planar imaging (EPI) sequence using the following parameters: repetition time (TR) = 2,000 ms, echo time (TE) = 30 ms, flip angle (FA) = 90°, matrix = 64 × 64, slice thickness = 4 mm, slice number = 33, and field of view (Fov) = 220 mm × 220 mm. The subjects were told to lie still in the scanner, eyes closed, but not to fall asleep. We collected 240 fMRI scans for each subject.

### Data Preprocessing

fMRI images were preprocessed with the statistical parametric mapping software package (SPM12)^1^ and the Gretna toolbox.^2^ Due to magnetic field instability, the first ten functional images were discarded and the remaining 230 scans from each subject were excluded in further analyses. Slice timing and realignment were first performed to correct for differences in acquisition time of slices and head motion, respectively. One patient without AVHs and one healthy control were excluded from further analyses due to excessive head motion (>3 mm translation or 3°rotation). fMRI images were then normalized into standard MNI space with a T1 unified segmentation strategy and then spatially smoothed with a Gaussian kernel filter of 6 mm full-width half-maximum (FWHM). After temporally detrending, nuisance signals including head motion profiles (using Friston-24 parameters), as well as signals from the white matter and cerebrospinal fluid (CSF) were regressed out. Since previous studies were controversial on whether the global signal should be regressed out, two preprocessing strategies were used, and we investigated functional connectivity both with and without global signal regression. Then, a band-pass filter was applied to remove low-frequency (<0.01 HZ) drift and high-frequency (>0.1 HZ) physiological noises. Finally, data scrubbing was performed to reduce the impact of head motion on the fMRI data. In specific, frames with frame-wise displacement (FD) greater than 0.5 were interpolated with data from one previous time point and two subsequent time points.

### Dynamic Functional Connectivity Analysis

Dynamic functional connectivity analysis was performed by using a sliding-window algorithm with the DynamicBC toolbox.^3^ After preprocessing, the brain was parcellated into 246 regions of interest (ROIs) according to the Brainnetome atlas.^4^ Mean time course of each ROI was obtained and we then investigated whole-brain functional connectivity by calculating the correlation between time courses of any pair of ROIs within each time window. In specific, functional connectivity within a time window whose length was 50 TR was first calculated. This window was then slid and functional connectivity matrix within each of a series of consecutive windows was obtained. Two different settings of the overlap between two neighboring windows were adopt in the current study. We first used the default setting of 0.6 (according to a step of 20 TR) provided by the DynamicBC toolbox. This step has also been conducted in previous studies (Shakil et al., 2016; Wei L. et al., 2018; Guo et al., 2020). Totally 10 time windows under this setting were obtained. In addition, another setting of the overlap (0.9) and obtained 37 time windows were also employed, benefiting with a better time resolution for researchers to investigate the dynamics in functional connectivity. In addition to dynamic functional connectivity matrices, a static functional connectivity matrix for each subject was also calculated by setting the window width to be 230 TR. Non-parametric permutation test was used to compare the static and dynamic functional connectivity maps in AVHs patients, non-AVHs patients, and healthy subjects. Bonferroni correction was used to correct for multiple comparison.

### Dynamic Functional Connectivity States

Dynamic functional connectivity states were obtained by the k-means clustering algorithms. The distance between two brain functional connectivity patterns was measured by the correlation between them. The maximum number of states was set as 10, and the optimal number of states then automatically estimated by the DynamicBC toolbox which averaged the optimal number of states estimated according to silhouette, Calinski-Harabasz, and Davies-Bouldin values.

## Results

### Static Functional Connectivity

**FIGURE 1:**
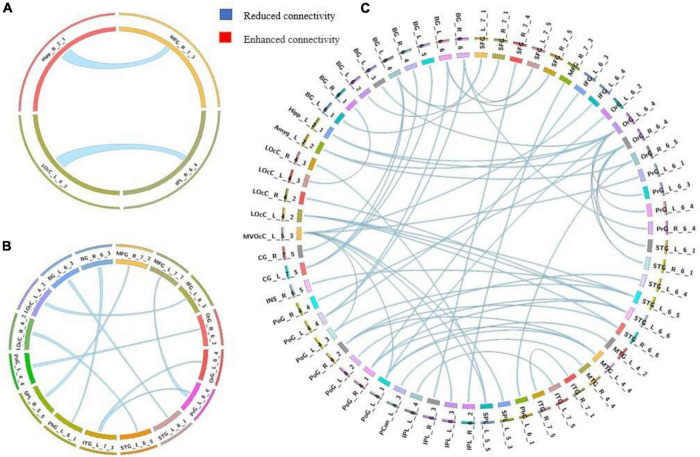
Group differences in static functional connectivity without regressing out global signal. **(A)** AVHs group compared with non-AVHs group; **(B)** non-AVHs group compared with NC group; **(C)** AVHs group compared with NC group. Increased connectivity is shown in red and decreased connectivity is shown in blue.

**FIGURE 2:**
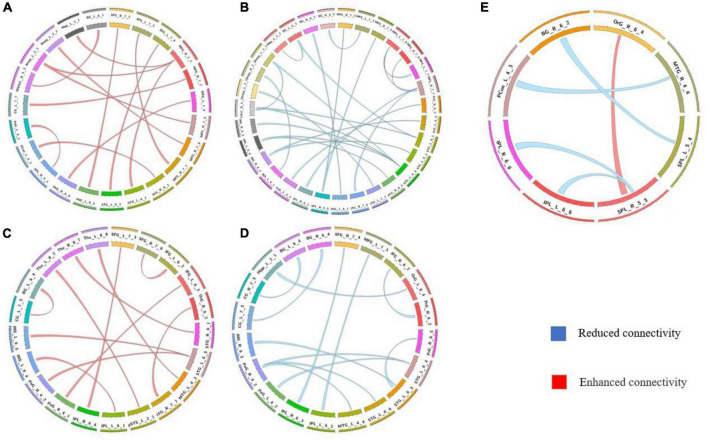
Comparison of the static functional connectivity of the three groups after global signal regression. **(A)** Static functional connections with enhanced connectivity in the non-AVHs group compared with the NC group; **(B)** Static functional connections with reduced connectivity in the non-AVHs group compared to the NC group; **(C)** Static connections with enhanced connectivity in AVHs group compared with NC group; **(D)** Static connections with reduced connectivity in AVHs group compared with NC group; **(E)** Compared with non-AVHs group, static connections with significant differences in AVHs group. Red represents increased connectivity and blue represents decreased connectivity.

We used a permutation test to compare static functional connectivity patterns among AVHs patients, non-AVHs patients, and healthy subjects. Without global signals regression, there were 2 connections with significant difference between the AVHs group and the non-AVHs group, 59 connections with significant difference between the AVHs group and the NC group, and 9 connections with significant difference between the non-AVHs group and the NC group (*p* < 0.05, Bonferroni corrected; Table 2 and Figure 1). With global signal regression, there were 5 connections with significant difference between AVHS and non-AVHs group, 23 connections with significant difference between AVHs and NC group, and 36 connections with significant difference between non-AVHs and NC group (*p* < 0.05, Bonferroni corrected; Table 3 and Figure 2). Although the between group differences with and without global signal regression consistently suggested mainly reduced static functional connectivity in patients with AVHs, compared to SZ patients without AVHs. Interestingly, without global signal regression, patients with AVHs only demonstrated reduced static functional connectivity compared with the other two groups. The involvement of global signal regression seemed to result in more enhanced connectivity in the AVHs group when compared with patients without AVHs and healthy controls.

**TABLE 2:** Differences in static functional connectivity without global signal regression.

AVHs and non-AVHs		AVHs and NC						Non-AVHs and NC	
Region 1	Region 2	Region 1	Region 2	Region 1	Region 2	Region 1	Region 2	Region 1	Region 2
IPL_R_6_4	LOcC_L_4_2	OrG_L_6_4	PrG_L_6_3	STG_R_6_6	PoG_R_4_2	OrG_L_6_4	Hipp_L_2_1	MFG_L_7_7	PrG_L_6_4
MFG_R_7_3	Hipp_R_2_1	OrG_R_6_4	PrG_L_6_4	MTG_L_4_4	PoG_R_4_2	OrG_R_6_4	Hipp_L_2_1	OrG_L_6_4	ITG_L_7_3
–	–	OrG_L_6_4	STG_L_6_1	STG_L_6_5	PoG_L_4_3	SFG_L_7_1	BG_L_6_1	PrG_L_6_4	ITG_L_7_3
–	–	OrG_R_6_4	STG_L_6_1	OrG_L_6_2	PoG_L_4_4	LOcC_L_4_3	BG_R_6_1	MFG_R_7_2	SPL_R_5_5
–	–	OrG_L_6_4	STG_R_6_1	OrG_R_6_4	PoG_R_4_4	SFG_L_7_1	BG_L_6_2	OrG_R_6_2	PoG_L_4_4
–	–	OrG_R_6_4	STG_R_6_1	STG_L_6_5	INS_R_6_5	OrG_L_6_4	BG_L_6_3	STG_L_6_5	LOcC_R_4_2
–	–	ITG_R_7_1	ITG_R_7_5	IFG_L_6_4	MVOcC_L_5_3	SFG_R_7_1	BG_L_6_4	IFG_L_6_3	LOcC_L_4_2
–	–	OrG_R_6_5	PhG_L_6_1	PrG_L_6_1	MVOcC_L_5_3	SFG_R_7_5	BG_L_6_4	STG_L_6_1	BG_L_6_3
–	–	MFG_R_7_3	IPL_R_6_2	SPL_L_5_3	MVOcC_L_5_3	CG_L_7_5	BG_L_6_4	PhG_L_6_1	BG_R_6_3
–	–	SFG_R_7_4	IPL_R_6_3	SPL_L_5_5	MVOcC_L_5_3	CG_R_7_5	BG_L_6_4	–	–
–	–	ITG_L_7_5	IPL_L_6_4	IPL_L_6_3	MVOcC_L_5_3	CG_L_7_5	BG_R_6_4	–	–
–	–	MTG_R_4_4	PCun_L_4_3	PoG_L_4_1	MVOcC_L_5_3	CG_R_7_5	BG_R_6_4	–	–
–	–	OrG_L_6_4	PoG_L_4_1	PoG_L_4_3	MVOcC_L_5_3	CG_L_7_5	BG_L_6_5	–	–
–	–	MTG_L_4_2	PoG_L_4_1	STG_L_6_5	LOcC_L_4_2	SFG_L_7_5	BG_L_6_6	–	–
–	–	MTG_L_4_4	PoG_L_4_1	STG_L_6_6	LOcC_L_4_2	SFG_R_7_5	BG_L_6_6	–	–
–	–	IFG_L_6_3	PoG_R_4_1	STG_R_6_6	LOcC_L_4_2	PoG_L_4_4	BG_L_6_6	–	–
–	–	STG_L_6_6	PoG_R_4_1	STG_L_6_6	LOcC_R_4_2	SFG_L_7_5	BG_R_6_6	–	–
–	–	MTG_L_4_4	PoG_R_4_1	SPL_L_5_5	LOcC_R_4_3	PrG_R_6_4	BG_R_6_6	–	–
–	–	STG_L_6_6	PoG_L_4_2	OrG_L_6_4	Amyg_L_2_2	PoG_L_4_4	BG_R_6_6	–	–
–	–	STG_L_6_4	PoG_R_4_2	OrG_R_6_5	Amyg_L_2_2	–	–	–	–

**TABLE 3:** Differences in static functional connectivity with global signal regression.

AVHs and non-AVHs		AVHs and NC				Non-AVHs and NC			
Region 1	Region 2	Region 1	Region 2	Region 1	Region 2	Region 1	Region 2	Region 1	Region 2
OrG_R_6_4	SPL_R_5_5	SFG_R_7_6	IFG_L_6_2	pSTS_L_2_1	Tha_L_8_2	MFG_L_7_6	MFG_L_7_7	MFG_R_7_2	CG_L_7_7
SPL_R_5_5	IPL_L_6_6	IFG_R_6_2	PrG_R_6_2	STG_R_6_1	Tha_R_8_7	SFG_L_7_3	MFG_R_7_7	STG_L_6_1	LOcC_R_4_2
SPL_R_5_5	IPL_R_6_6	PrG_R_6_5	STG_L_6_5	MTG_L_4_3	Tha_L_8_8	MFG_L_7_6	MFG_R_7_7	STG_L_6_5	LOcC_R_4_2
MTG_R_4_4	PCun_L_4_3	OrG_R_6_2	ITG_R_7_7	–	–	MFG_R_7_6	MFG_R_7_7	MFG_R_7_1	Amyg_L_2_1
SPL_L_5_4	BG_R_6_2	MFG_L_7_3	IPL_L_6_2	–	–	MFG_R_7_1	STG_L_6_1	MFG_R_7_5	Amyg_L_2_1
–	–	SFG_R_7_4	IPL_R_6_3	–	–	SFG_L_7_4	STG_L_6_2	ITG_L_7_3	Amyg_L_2_1
–	–	SFG_L_7_3	IPL_R_6_4	–	–	MFG_R_7_7	ITG_R_7_6	INS_L_6_1	Amyg_R_2_1
–	–	IFG_L_6_3	PoG_R_4_1	–	–	SFG_R_7_2	PhG_L_6_5	MFG_R_7_6	Amyg_L_2_2
–	–	STG_L_6_4	PoG_L_4_2	–	–	MFG_L_7_4	PhG_R_6_6	STG_L_6_5	Amyg_L_2_2
–	–	STG_L_6_4	PoG_R_4_2	–	–	MFG_R_7_2	SPL_R_5_5	LOcC_R_4_2	Amyg_L_2_2
–	–	STG_L_6_5	PoG_R_4_2	–	–	MFG_R_7_5	SPL_R_5_5	MFG_R_7_1	Hipp_L_2_1
–	–	STG_L_6_6	PoG_R_4_2	–	–	MFG_R_7_7	IPL_R_6_2	MVOcC_R_5_5	BG_L_6_1
–	–	MTG_L_4_4	PoG_R_4_2	–	–	OrG_R_6_2	PCun_L_4_3	STG_L_6_1	BG_L_6_3
–	–	IPL_L_6_1	INS_L_6_4	–	–	SPL_R_5_1	PoG_L_4_2	Hipp_L_2_2	BG_L_6_3
–	–	STG_L_6_5	INS_L_6_5	–	–	OrG_R_6_2	PoG_L_4_4	PhG_L_6_1	BG_R_6_3
–	–	STG_L_6_5	INS_R_6_5	–	–	PrG_L_6_5	INS_R_6_2	MFG_L_7_3	BG_R_6_5
–	–	OrG_L_6_4	Hipp_L_2_1	–	–	STG_L_6_3	INS_R_6_2		
–	–	CG_L_7_5	BG_L_6_4	–	–	STG_L_6_5	INS_L_6_5		
–	–	CG_R_7_5	BG_L_6_4	–	–	STG_R_6_5	INS_L_6_5		
–	–	CG_L_7_5	BG_R_6_4	–	–	STG_L_6_5	INS_R_6_5		
									

### Dynamics of Functional Connectivity

**FIGURE 3:**
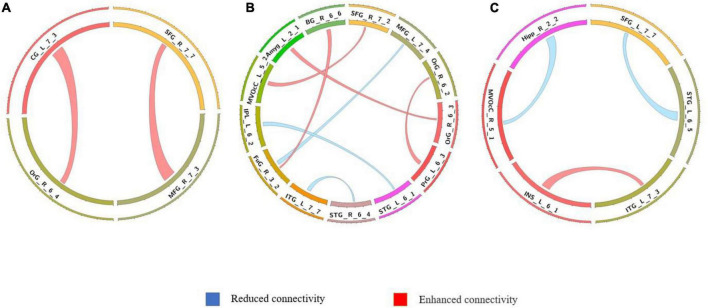
Comparison of dynamic differences in functional connectivity without global signal regression (overlap = 0.6). **(A)** AVHs group compared with the non-AVHs group; **(B)** AVHs group compared with the NC group; **(C)** non-AVHs group compared with the NC group. Increased connectivity shown in red and decreased shown in blue.

**FIGURE 4:**
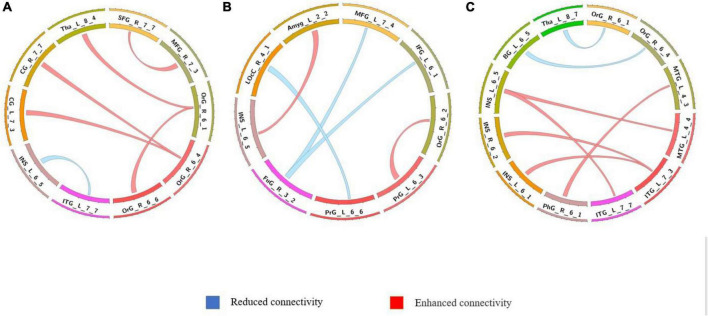
Comparison of dynamic functional connectivity without global signal regression (overlap = 0.9). **(A)** AVHs group compared with the non-AVHs group; **(B)** AVHs group compared with the NC group; **(C)** non-AVHs group compared with the NC group. Increased connectivity was shown in red and decreased shown in blue.

**FIGURE 5:**
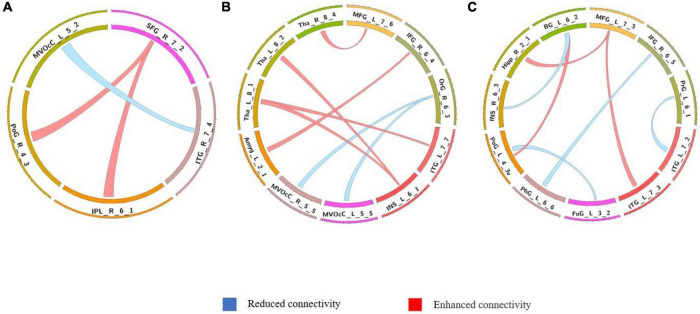
Comparison of dynamic differences in functional connectivity with global signal regression (overlap = 0.6). **(A)** AVHs group compared with the non-AVHs group; **(B)** AVHs group compared with the NC group; **(C)** non-AVHs group compared with the NC group.

**FIGURE 6:**
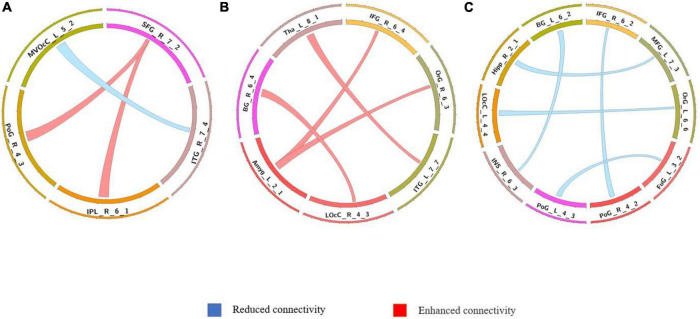
Comparison of dynamic differences in functional connectivity with global signal regression (overlap = 0.9). **(A)** AVHs group compared with the non-AVHs group; **(B)** AVHs group compared with the NC group; **(C)** non-AVHs group compared with the NC group. Increased connectivity shown in red and decreased shown in blue.

The differences in the dynamics of whole-brain functional connectivity between the three groups were accessed by comparing the variance of dynamic functional connectivity matrices. For each connection, we calculated the variance of this connection over all the sliding windows to quantify its variability. Then a non-parametric permutation test was used to compare dynamics of functional connectivity of the three groups (*P* < 0.05; Bonferroni correction; Tables 4–7 and Figures 3–6). As shown in Figures 3–6, SZ patients with and without AVHs showed differences in functional connectivity compared with the healthy controls. The AVHs group consistently demonstrated enhanced dynamics in functional connectivity compared with the non-AVHs group under different settings of overlap between neighboring windows, as well as with different data preprocessing strategies.

**TABLE 4:** Significant differences in variances of the dynamic functional connectivity matrices obtained without regressing out global signal (overlap = 0.6).

AVHs and non-AVHs		AVHs and NC		Non-AVHs and NC	
Region 1	Region 2	Region 1	Region 2	Region 1	Region 2
SFG_R_7_7	MFG_R_7_3	OrG_R_6_2	PrG_L_6_3	SFG_L_7_7	STG_L_6_5
OrG_R_6_4	CG_L_7_3	STG_R_6_4	ITG_L_7_7	ITG_L_7_3	INS_L_6_1
–	–	MFG_L_7_4	FuG_R_3_2	MVOcC_R_5_1	Hipp_R_2_2
–	–	STG_L_6_1	IPL_L_6_2	–	–
–	–	SFG_R_7_2	MVOcC_L_5_2	–	–
–	–	OrG_R_6_3	Amyg_L_2_1	–	–
–	–	FuG_R_3_2	BG_R_6_6	–	–

**TABLE 5:** Significant differences in variances of the dynamic functional connectivity matrices obtained without regressing out global signal (overlap = 0.9).

AVHs and non-AVHs		AVHs and NC		Non-AVHs and NC	
Region 1	Region 2	Region 1	Region 2	Region 1	Region 2
SFG_R_7_7	MFG_R_7_3	OrG_R_6_2	PrG_L_6_3	MTG_L_4_3	PhG_R_6_1
OrG_R_6_1	OrG_R_6_6	MFG_L_7_4	FuG_R_3_2	ITG_L_7_3	INS_L_6_1
ITG_L_7_7	INS_L_6_5	IFG_L_6_1	FuG_R_3_2	ITG_L_7_3	INS_R_6_2
OrG_R_6_4	*CG_L_7_3*	PrG_L_6_6	LOcC_R_4_1	MTG_L_4_4	INS_L_6_5
OrG_R_6_4	CG_R_7_7	INS_L_6_5	Amyg_L_2_2	ITG_L_7_7	INS_L_6_5
OrG_R_6_1	Tha_L_8_4	–	–	OrG_R_6_4	BG_L_6_5
–	–	–	–	OrG_R_6_1	Tha_L_8_7
					

**TABLE 6:** Significant differences in variances of the dynamic functional connectivity matrices obtained with global signal regression (overlap = 0.6).

AVHs and non-AVHs		AVHs and NC		Non-AVHs and NC	
Region 1	Region 2	Region 1	Region 2	Region 1	Region 2
SFG_R_7_2	IPL_R_6_1	OrG_R_6_3	MVOcC_L_5_5	PrG_L_6_1	ITG_L_7_2
SFG_R_7_2	PoG_R_4_3	OrG_R_6_3	MVOcC_R_5_5	MFG_L_7_3	ITG_L_7_3
ITG_R_7_4	MVOcC_L_5_2	IFG_R_6_4	Amyg_L_2_1	IFG_R_6_5	PhG_L_6_6
–	–	ITG_L_7_7	Tha_L_8_1	FuG_L_3_2	PoG_L_4_3
–	–	INS_L_6_1	Tha_L_8_1	MFG_L_7_3	Hipp_R_2_1
–	–	INS_L_6_1	Tha_L_8_2	PoG_L_4_3v	BG_L_6_2
–	–	MFG_L_7_6	Tha_R_8_4	INS_R_6_3	BG_L_6_2

**TABLE 7:** Significant differences in variances of the dynamic functional connectivity matrices obtained with global signal regression (overlap = 0.9).

AVHs and non-AVHs		AVHs and NC		Non-AVHs and NC	
Region 1	Region 2	Region 1	Region 2	Region 1	Region 2
SFG_R_7_2	IPL_R_6_1	IFG_R_6_4	Amyg_L_2_1	IFG_R_6_2	PoG_R_4_2
SFG_R_7_2	PoG_R_4_3	OrG_R_6_3	Amyg_L_2_1	FuG_L_3_2	PoG_L_4_3
ITG_R_7_4	MVOcC_L_5_2	LOcC _R_4_3	BG_R_6_4	OrG_L_6_6	LOcC_L_4_4
–	–	ITG_L_7_7	Tha_L_8_1	MFG_L_7_3	Hipp_R_2_1
–	–	–	–	INS_R_6_3	BG_L_6_2

### Dynamic Functional Connectivity States

**FIGURE 7:**
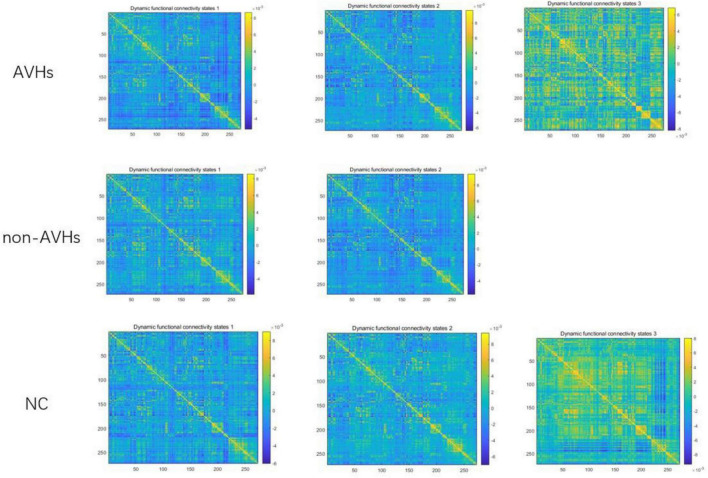
Patterns of dynamic functional connectivity states in the AVHs, non-AVHs groups and NC groups without global signal regression (overlap = 0.6).

The optimal number of dynamic function connection states estimated by k-means algorithm is 3 for AVHs group, 2 for non-AVHs group, and 3 for NC group. Figure 7 shows the spatial patterns of these three groups of brain states. From Figure 7 we observed that the number of optimal states estimated in the AVH group was higher than that in the non-AVH group. This observation is also robust under different setting of overlap (overlap = 0.9) seen in Supplementary Figure 1–3, suggesting that the resting-sate functional connectivity of the AVHs group seems to be alternating among more brain states than that of the non-AVHs group. This finding is also in line with our observation of increased variance of dynamic functional connectivity matrices, suggesting enhanced brain dynamics in patients with AVHs.

## Discussion

In this study, we examined the differences in static FC. Importantly, we also examined the differences in dynamic FC. The results showed that compared with NC, both AVHs group and non-AVHs group showed only decreased static functional connectivity when without global signal regression. Instead, resulting in increasing connectivity between the two groups with global signal regression, most of the static functional connectivity in the AVHs group showed decreased connectivity compared to the non-AVHs group.

In general, The AVHs group consistently demonstrated enhanced dynamics in functional connectivity compared with the non-AVHs group under different settings of overlap between neighboring windows, as well as with different data preprocessing strategies (with/without global regression; overlap=0.6/0.9). These findings suggested that patients with AVHs are characterized by reduced strength of static functional connectivity, accompanied with enhanced dynamics of functional connectivity.

From the analysis of functional connectivity, we found that auditory hallucinations in patients with schizophrenia may be related to abnormal functional connectivity among the frontal lobe, temporal regions and parietal regions. In fact, auditory verbal hallucinations are considered to be a disease caused by the patient’s inability to recognize the internal language generated by the brain (Anthony, 2004; Zhuo et al., 2021). This loss of cognitive internal language ability is highly correlated with the frontal cortex of speech generation and the temporal and parietal regions of sensory processing (Frith, 2005). With the proposal of “abnormal connection hypothesis,” the connection between these language brain regions is becoming ever more important in the study of auditory hallucinations (Li et al., 2017). In this study, we studied and compared the whole brain functional connectivity and patterns of schizophrenia patients with and without auditory hallucinations and normal subjects.

Compared with non-AVHs group, the AVHs group were characterized by significantly enhanced static functional connectivity among Frontal Gyrus, Inferior Parietal Lobule, and Hippocampus. The above brain areas are related to language acquisition and language understanding, which further imply that there were abnormalities in the connection of brain language regions in auditory hallucinations (Vercammen et al., 2010; Clos et al., 2014). FMRI study found that the decrease of functional connection between temporal and parietal lobes in patients with AVHs was positively correlated with the severity of auditory hallucinations (Vercammen et al., 2010). This study also revealed that many of the connections demonstrated increased variability in the AVHs group compared with the non-AVHs group. The above findings are consistent with some research results (Cui et al., 2017a; Barber et al., 2021), indicating that AVHs have something to do with dysfunction of the regions involving speech imagery, production, and monitoring, and schizophrenia patients with AVHs showed deficit communication between the brain network.

Our findings are a little different from previous results. These contradictory results may be related to the heterogeneity of schizophrenia and the sample size. The characteristic of this study is to directly compare AVHs and non-AVHs and reveal the pathophysiological basis of auditory hallucinations intuitively.

There are several potential limitations of the current study. First, schizophrenia patients with AVHs may hallucinate or fall asleep during the scan, thus losing control over the design of the resting state. Second, small sample sizes may lead to reduced reliability of inter-group differences. The correlation strength between auditory hallucination phenotype and language-related regions in schizophrenia patients has yet to be studied by expanding the sample size.

## Conclusion

The results showed that the connectivity of most static functional connections was significantly reduced, and the dynamic of functional connections was significantly increased in the AVHs group under the two preprocessing strategies. In a cluster analysis of the two groups based on the dynamic nature of functional connectivity, schizophrenia patients with AVHs shifted between more brain states. The results suggest that significant changes in functional connectivity of the brain may contribute to the study of pathophysiological mechanisms of schizophrenia patients with AVHs and the diagnosis of AVHs.

## Data Availability Statement

The raw data supporting the conclusions of this article will be made available by the authors, without undue reservation.

## Ethics Statement

The experiment was approved by the Ethics Committee of the Fourth Military Medical University. The patients/participants provided their written informed consent to participate in this study. Written informed consent was obtained from the individual(s) for the publication of any potentially identifiable images or data included in this article.

## Author Contributions

YaZ and PH conceptualized and designed the research. SQ and HL collected demographics and MRI data. JW, XL, and PH analyzed MRI data and undertook statistical analysis with WL. PH, JW, YnZ, WH, YC, YW, and MY wrote the first draft. PH, JG, XH, XZ, JW, WZ, and YaZ contributed to the final manuscript including editing figures, tables, and format. All authors critically reviewed the content and approved the final version for publication.

## Conflict of Interest

The authors declare that the research was conducted in the absence of any commercial or financial relationships that could be construed as a potential conflict of interest.

## Publisher’s Note

All claims expressed in this article are solely those of the authors and do not necessarily represent those of their affiliated organizations, or those of the publisher, the editors and the reviewers. Any product that may be evaluated in this article, or claim that may be made by its manufacturer, is not guaranteed or endorsed by the publisher.

## Footnotes

1
http://www.fil.ion.ucl.ac.uk/spm/software/spm12/

2
https://www.nitrc.org/projects/gretna/

3
https://guorongwu.github.io/DynamicBC/

4
http://atlas.brainnetome.org/brainnetome.html
