# Supplementary figures and images for: Distinct Brain Dynamic Functional Connectivity Patterns in Schizophrenia Patients With and Without Auditory Verbal Hallucinations
Source: Front Hum Neurosci. 2022 Apr 7;16:838181. doi: 10.3389/fnhum.2022.838181 (PMC9023234; doi:10.3389/fnhum.2022.838181)

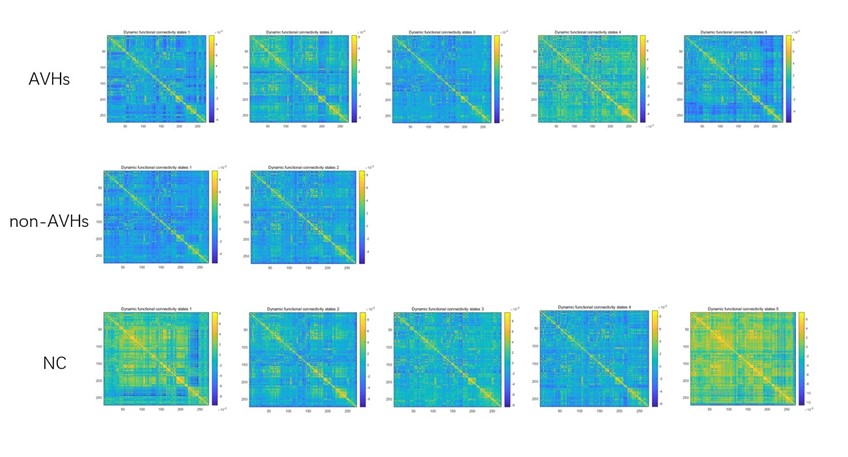

Supplement: Supplementary file 1 [file Image_1.JPEG]

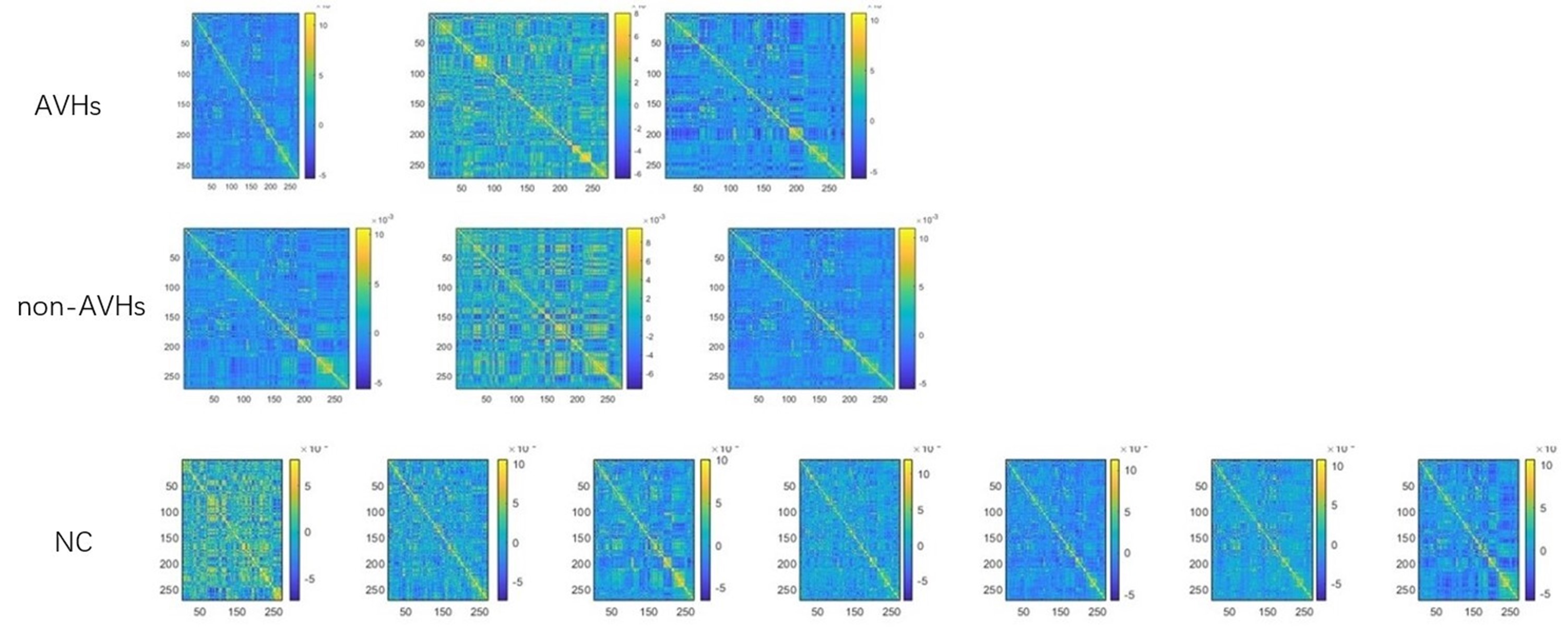

Supplement: Supplementary file 2 [file Image_2.JPEG]

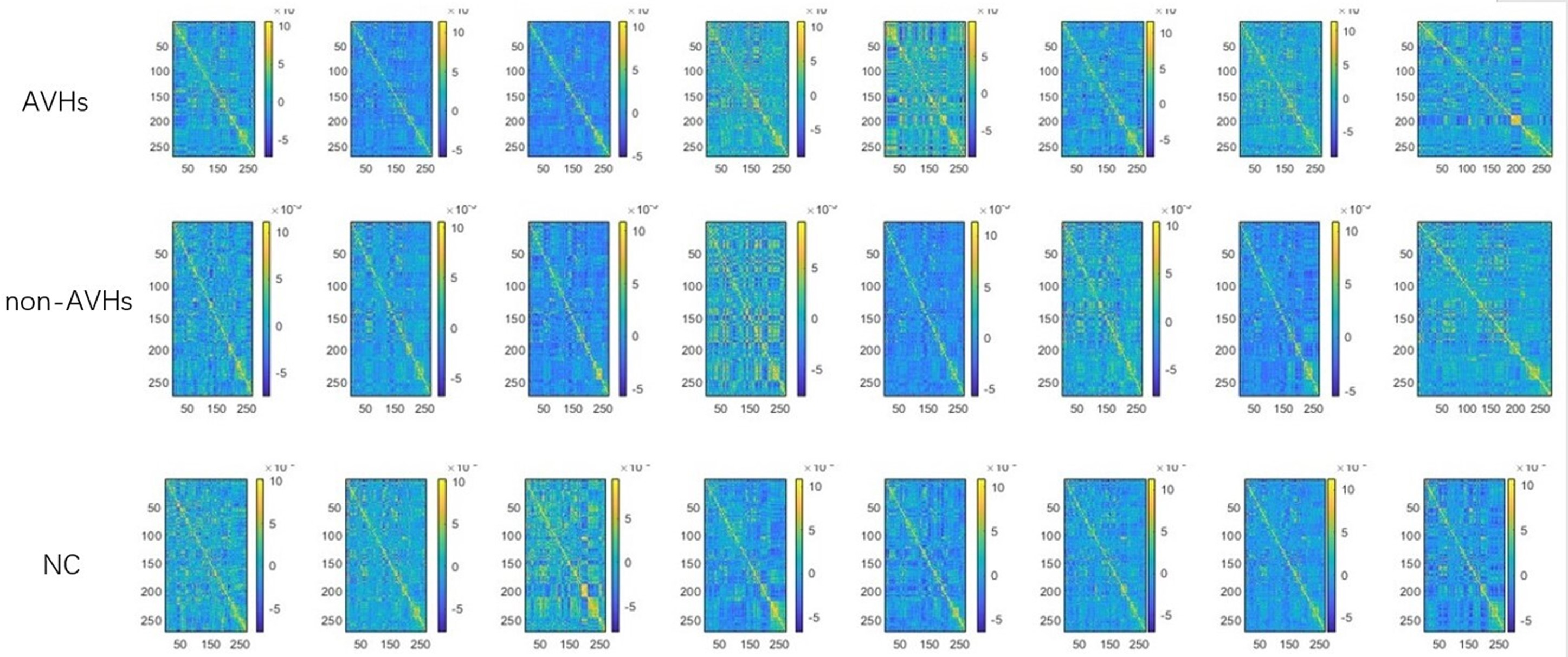

Supplement: Supplementary file 3 [file Image_3.JPEG]
